# Framework for patient-specific simulation of hemodynamics in heart failure with counterpulsation support

**DOI:** 10.3389/fcvm.2022.895291

**Published:** 2022-08-01

**Authors:** Mattia Arduini, Jonathan Pham, Alison L. Marsden, Ian Y. Chen, Daniel B. Ennis, Seraina A. Dual

**Affiliations:** ^1^Department of Radiology, Stanford University, Palo Alto, CA, United States; ^2^Mechanical Engineering, Stanford University, Palo Alto, CA, United States; ^3^Department of Bioengineering, Stanford University, Palo Alto, CA, United States; ^4^Department of Pediatrics, Stanford University, Palo Alto, CA, United States; ^5^Cardiovascular Institute, Stanford University, Palo Alto, CA, United States; ^6^Division of Medicine (Cardiology), Veterans Affairs Health Care System, Palo Alto, CA, United States; ^7^Division of Radiology, Veterans Affairs Health Care System, Palo Alto, CA, United States

**Keywords:** HF, patient-specific, hemodynamics, lumped parameter, counterpulsation, McKibben, soft robot

## Abstract

Despite being responsible for half of heart failure-related hospitalizations, heart failure with preserved ejection fraction (HFpEF) has limited evidence-based treatment options. Currently, a substantial clinical issue is that the disease etiology is very heterogenous with no patient-specific treatment options. Modeling can provide a framework for evaluating alternative treatment strategies. Counterpulsation strategies have the capacity to improve left ventricular diastolic filling by reducing systolic blood pressure and augmenting the diastolic pressure that drives coronary perfusion. Here, we propose a framework for testing the effectiveness of a soft robotic extra-aortic counterpulsation strategy using a patient-specific closed-loop hemodynamic lumped parameter model of a patient with HFpEF. The soft robotic device prototype was characterized experimentally in a physiologically pressurized (50–150 mmHg) soft silicone vessel and modeled as a combination of a pressure source and a capacitance. The patient-specific model was created using open-source software and validated against hemodynamics obtained by imaging of a patient (male, 87 years, HR = 60 bpm) with HFpEF. The impact of actuation timing on the flows and pressures as well as systolic function was analyzed. Good agreement between the patient-specific model and patient data was achieved with relative errors below 5% in all categories except for the diastolic aortic root pressure and the end systolic volume. The most effective reduction in systolic pressure compared to baseline (147 vs. 141 mmHg) was achieved when actuating 350 ms before systole. In this case, flow splits were preserved, and cardiac output was increased (5.17 vs. 5.34 L/min), resulting in increased blood flow to the coronaries (0.15 vs. 0.16 L/min). Both arterial elastance (0.77 vs. 0.74 mmHg/mL) and stroke work (11.8 vs. 10.6 kJ) were decreased compared to baseline, however left atrial pressure increased (11.2 vs. 11.5 mmHg). A higher actuation pressure is associated with higher systolic pressure reduction and slightly higher coronary flow. The soft robotic device prototype achieves reduced systolic pressure, reduced stroke work, slightly increased coronary perfusion, but increased left atrial pressures in HFpEF patients. In future work, the framework could include additional physiological mechanisms, a larger patient cohort with HFpEF, and testing against clinically used devices.

## 1. Introduction

In heart failure (HF) the heart is unable to appropriately supply the receiving organs with blood, leading to over five million hospitalizations yearly (1–4). To date, we have limited evidence-based treatment options for patients with heart failure with preserved ejection fraction (HFpEF) even though they represent nearly half of the HF population (5). HFpEF is characterized by suboptimal filling of the LV during diastole due to lower compliance of the ventricular walls (6). The syndrome leads to higher LV pressure levels to obtain a certain LV volume than in healthy subjects (7). Consequently, patients experience dyspnea on exertion, reduced quality of life, and reduced life expectancy (6, 8).

Recent efforts have proposed multiple device-based treatment options such as the intra-atrial shunt device (9, 10), use of conventional left ventricular assist devices (11, 12) for atrial decompression, a valveless pulsatile left ventricular assist device (13), and a left atrial assist device (14). However, the development of treatment strategies for HFpEF is complicated by the heterogeneity in pathophysiologies and associated comorbidities (3, 15, 16). A recent study compared the above therapies for multiple cohorts of patients with HFpEF (17) and found that each treatment may be effective only in a sub-cohort of HFpEF. As an alternative to grouping patients, we aimed to present a framework for patient-specific testing of treatment strategies and use it to test the usefulness of aortic counterpulsation in HFpEF.

Counterpulsation is a treatment strategy often applied in HF with reduced ejection fraction (HFrEF), but understudied for its usefulness in HFpEF. Counterpulsation reduces afterload and systolic pressure, and increases coronary perfusion (18). Intra-aortic balloon pumps (IABPs) are the most commonly used form of counterpulsation; the device is placed in the descending aorta and directs diastolic blood flow toward the periphery (18). The risk factors of IABPs include vascular injury, embolic complications during deployment or removal, and bacterial infections (19), motivating a need for non-blood contacting extra-aortic actuation. The C-Pulse System (Nuwellis Inc., Eden Prairie, Mn) is an extra-aortic counterpulsation device for HF which has shown to reduce systolic pressure and afterload in clinical trials (20, 21). The hemodynamic benefits of counterpulsation, such as decreased systolic pressures and increased coronary filling, have previously been suggested in small cohorts to improve diastolic filling (22, 23). Potential benefits of counterpulsation in patients with HFpEF have not been previously assessed.

Patient-specific lumped parameter computational models provide a low-cost option for proof-of concept testing of innovative treatment options in interaction with the cardiovascular system. Lumped parameter computational models are efficient (24) and are particularly suitable for testing in heterogeneous patient populations as each component can be tuned to match anatomical and hemodynamic data in a patient-specific manner (25, 26). Given the heterogeneity of the HFpEF population, a patient-specific approach is particularly important to allow an extension to a patient cohort in the future.

**Figure 1:**
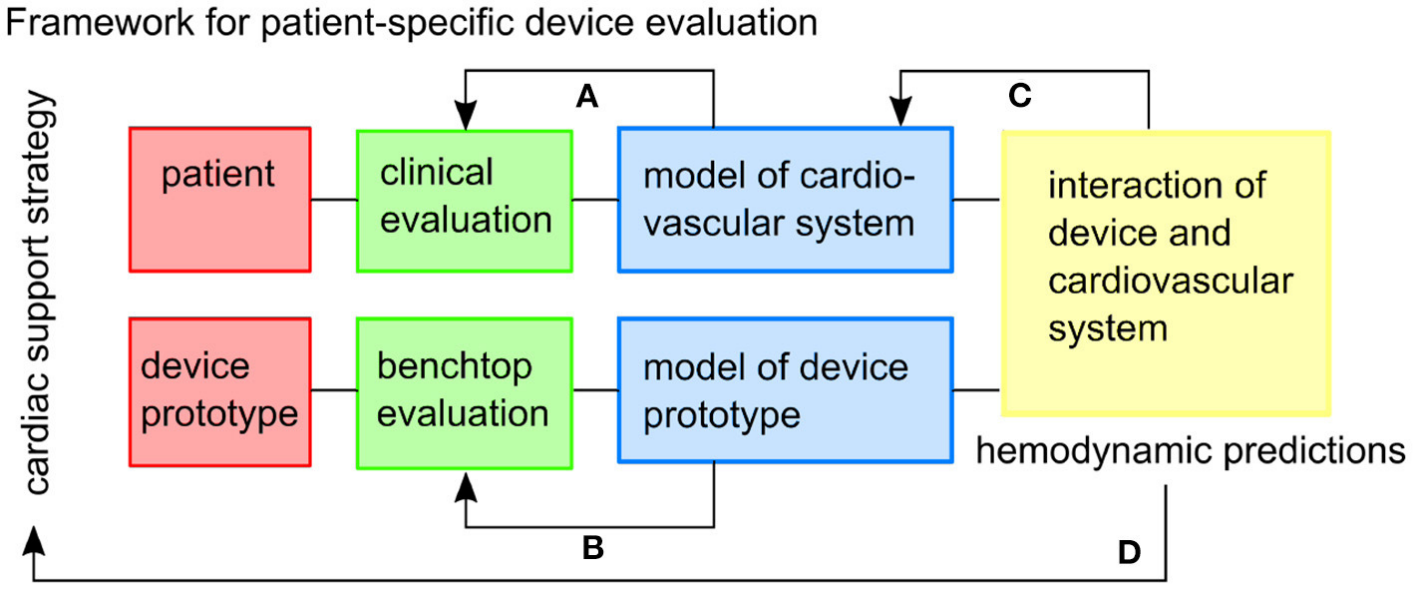
The framework envisioned for patient-specific testing of cardiac support strategies. Feedback within the framework includes: **(A)** the patient-specific cardiovascular model is tuned to the clinical data, **(B)** the model of the device prototype is informed by benchtop experiments, **(C)** the combined model might inform additional physiological mechanisms to be included in the cardiovascular model, **(D)** and hemodynamic predictions can inform the usefulness of the cardiac support strategy in the specific patient cohort.

We demonstrated and evaluated a framework (Figure 1) for prototyping a soft robotic device prototype and modeling its interaction with patient-specific HFpEF hemodynamics. An extra-aortic soft-robotic counterpulsation device prototype was characterized experimentally, as a part of this work. We assess the impact of this device prototype on lowering peak systolic pressures and increasing coronary filling using a closed-loop patient-specific lumped parameter model at different timings and actuation levels.

## 2. Materials and methods

### 2.1. Patient-specific lumped parameter model of HFpEF

We created a patient-specific lumped parameter model of the cardiovascular system tuned to the hemodynamics of a patient with HFpEF. The study was approved by the local IRB board. Aortic blood flows measurements were obtained by 2D phase contrast cardiac magnetic resonance imaging and blood pressures as upper arm cuff pressures within 1 h of acquisition (male, 87 years, HR = 60 bpm). The patient was selected based on high diastolic filling pressures assessed by echocardiography (Lat E/e' = 14.5, Med E/e' = 18.4 at/above 15), a criteria defined by the American Society of Echocardiography and the European Association of Cardiovascular Imaging in (27). We utilized an estimate of end-diastolic pressure of 16 mmHg based on Lat E/e' = 14.5, Med E/e' = 18.4, a conversion proposed by Ommen et al. (28), as no invasive pressure data was available.

**Figure 2:**
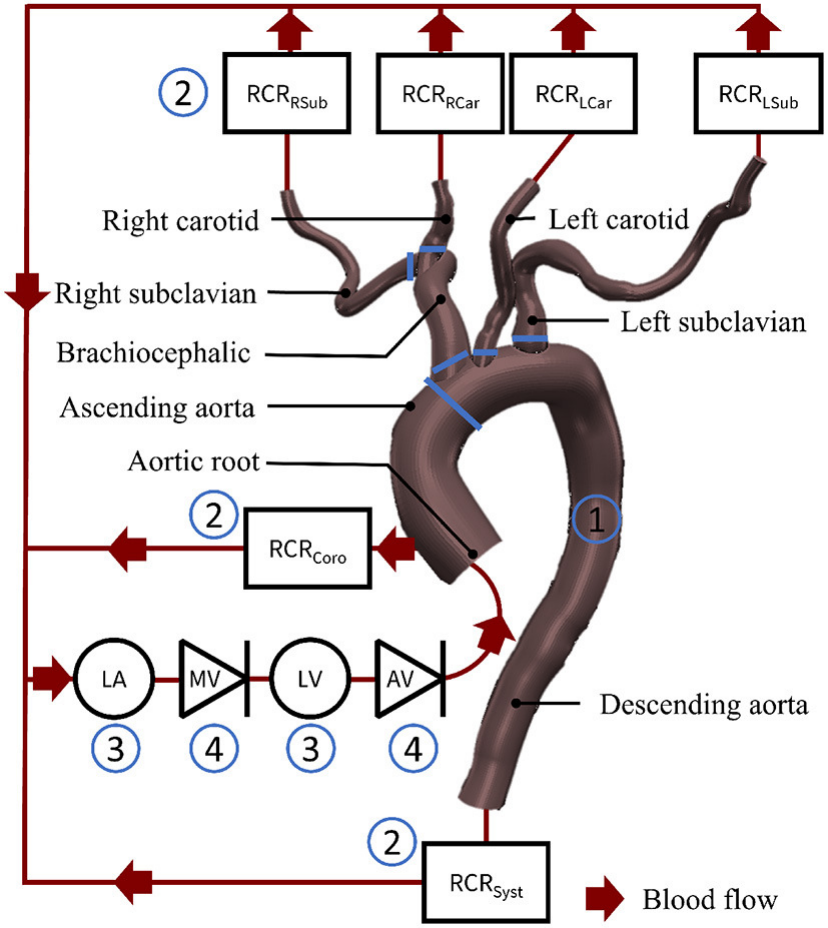
The lumped parameter model of the cardiovascular system, **(1)** is the aorta, **(2)** are the peripheral circulation elements, **(3)** are the chamber elements, **(4)** are the valve elements. LA, left atrium; MV, mitral valve; LV, left ventricle; AV, aortic valve; RCRRSub, resistance capacitance resistance element at the right subclavian artery; RCRRCar, resistance capacitance resistance element at the right carotid artery; RCRLCar, resistance capacitance resistance element at the left carotid artery; RCRLSub, resistance capacitance resistance element at the left subclavian artery; RCRCoro, resistance capacitance resistance element at the coronary arteries; RCRSyst, resistance capacitance resistance element of the systemic circulation.

The computational model consisted of the vessels of the upper and lower bodies, the left heart and the valves (Figure 2) connected as a closed-loop. Each component was represented as a combination of resistors R representing the pressure drop caused by viscous losses, capacitors C capturing the elastance of the vessels, and inductances L modeling the inertial component of blood.

Initial conditions of pressure, flow, and volume for the first cardiac cycle were estimated using a minimum squared error function. The function starts solving for the initial conditions based on values found in literature. A time-periodic state is reached after five cardiac cycles.

#### 2.1.1. The vessels

The lumped parameter model of the main arterial vessels (aortic arch) was created with the following steps. We first constructed an anatomic model using SimVascular (http://simvascular.github.io) (Point 1 in Figure 2) based on a computational tomography (CT) scan of the HFpEF patient. First, we drew pathlines along the centerlines of the arterial vessels of interest. We then segmented the CT scan by outlining each vessel lumen manually to produce 10 to 20 evenly spaced cross sections normal to the vessel centerlines (29). We then ran an automated script in SimVascular to calculate the resistances of each aortic branch vessel based on the average of its varying cross sectional area assuming Poiseuille flow (30). Each vessel element was reduced to three resistor sub-elements placed in series. At the end of each branch a resistor capacitor resistor (RCR) element tuned to achieve the patient's flow splits represented the distal vasculature (including the microvasculature) (Point 2 in Figure 2). We assumed rigid vessel walls (29, 31).

#### 2.1.2. The left heart

A time-dependent elastance model relating pressure to volume (Point 3 in Figure 2) represented the left ventricle and atrium of the heart and was presented by Colacino et al. (32). Diastole and systole were modeled through a passive and an active pressure component.

#### 2.1.3. The valves

The mitral valve (MV) and the aortic valve (AV) (Point 4 in Figure 2) were modeled as pressure-actuated valves (33). The modeled heart valves only allow the flow to go through when the pressure on the inlet side is higher than the pressure on the outlet side. The state *s*(*t*) of the valve changes between 0 and 1 with 0 being a fully closed valve.

### 2.2. Lumped parameter model of the soft robot

**Figure 3:**
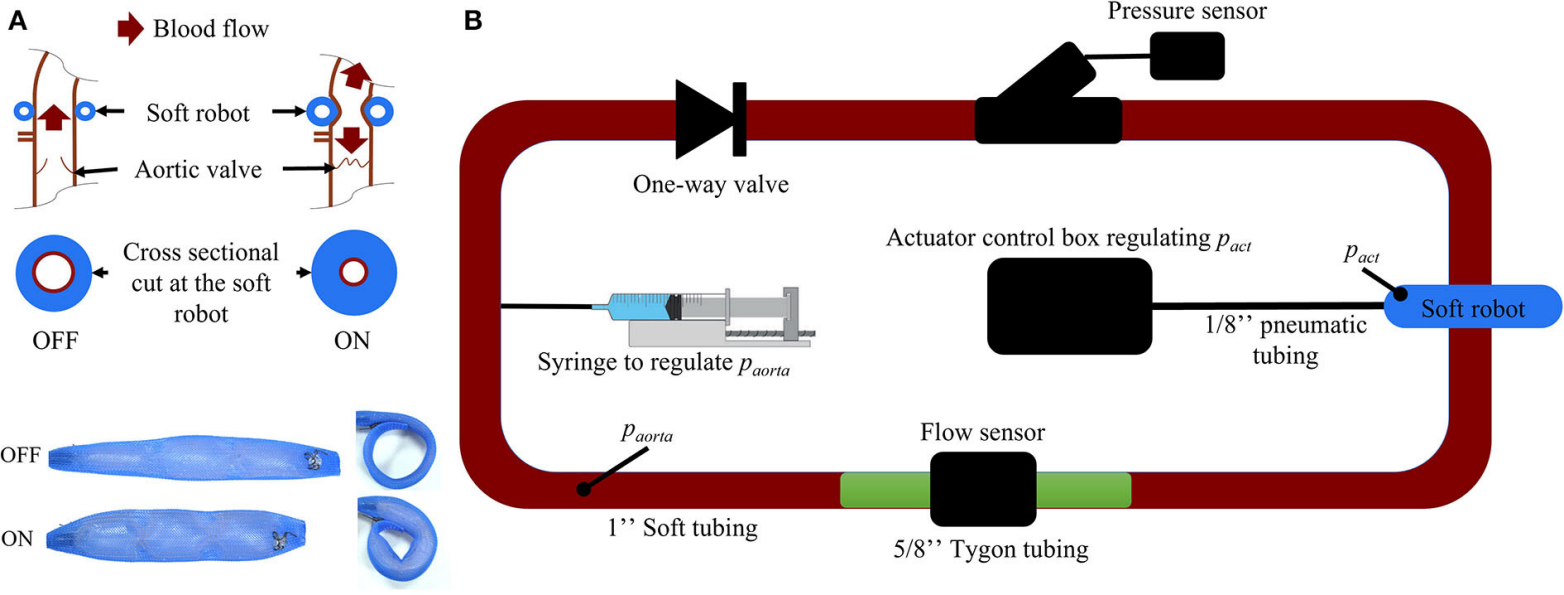
**(A)** Working mechanism of the soft robotic McKibben actuator on the ascending aorta. **(B)** Passive flow loop for the soft robot characterization [*p*_*aorta*_, pressure inside the passive loop (mmHg); *p*_*act*_, pressure inside the soft robot (psi)].

The prototype of the soft robot is based on the McKibben actuator principle and consists of a single non-elastic pneumatically actuated bladder in a mesh which is wrapped around the ascending aorta (Figure 3A). When injecting a user-determined pressure *p*_*act*_, the McKibben actuator will shorten circumferentially while thickening circumferentially and thus contract the aorta. The maximum width of the device prototype upon inflation is 12 mm. The inflation of the actuator induces a pressure on the ascending aorta, which in turn induces a change in volume inside the vessel. We used the capacitance-based approach proposed by Schampaert et al. to model the soft robot (34). The capacitor element receives an input by an experimentally defined pressure source *p*_*sr*_ composed of a contraction part followed by a relaxation part (Equation 1) mimicking the soft robot's effect on a vessel (Section 2.2.2).

#### 2.2.1. Analytical definition

The parameters defining the soft robot actuation were the duration of the contraction *t*_*act*_, the delay with respect to the beginning of the cardiac cycle δ*t*_*act*_ (measured as R-wave of the electrocardiogram), the pressure inside the actuator *p*_*act*_, and the pressure inside the aorta *p*_*aorta*_. The duration of the actuator contraction was always 300 ms, such that for delays above 700 ms the actuator only deflated at the beginning of the next cycle. The duration of the contraction of the soft robot was *t*_*on*_ while *t*_*off*_ was the duration of a full actuation cycle including the relaxation. The times *t*_*on*_ and *t*_*off*_ varied for different *p*_*act*_ and were determined experimentally (Supplementary material). Our experimental results confirmed that a higher *p*_*act*_ leads to higher peak pressures while the peak pressure is reduced for higher *p*_*aorta*_.


(1) $$p_{sr}(t)=\left\{ \begin{array}{l} \begin{array}{cc} \frac{p_{aorta}(t_{norm})}{1-g_{act}(p_{aorta},p_{act})}*(1-g_{act} & \text{for }0\leq t_{norm}\leq t_{on} \end{array}\\ \left(p_{aorta},p_{act})*e^{-k_{1}*t_{norm}}\right)\\ \begin{array}{cc} c_{2}*e^{-k_{2}*(t_{norm}-t_{on})}+c_{2} & \text{for }t_{on}<t_{norm}\leq t_{off} \end{array}\\ \begin{array}{cc} 0 & \text{else} \end{array} \end{array}\right.$$

here $t_{norm}=t-\left\lfloor \frac{t-\delta t_{act}*HR}{60}\right\rfloor *\frac{60}{HR}-\delta t_{act}$ is the time t normalized with respect to the cycle and actuation delay. *g*_*act*_(*p*_*aorta*_, *p*_*act*_) is an experimental coefficient. Time coefficients *k*_1_ and *k*_2_ were determined experimentally and are equal to 13.04 and 34.66 respectively. δ*t*_*act*_ is the delay of the soft robot actuation. Coefficients *c* and *d* ensure continuity between the contraction and relaxations functions at moments *t*_*on*_ and *t*_*off*_.


(2) $$\begin{array}{l} g_{act}(p_{act},p_{aorta})=a_{1}\;*\;p_{aorta}^{2}+a_{2}\;*\;p_{aorta}+a_{3}\;*\;p_{act}\\ \;+a_{4}+a_{5}\;*\;p_{aorta}\;*\;p_{act} \end{array}$$

where the coefficients *a*_1_, *a*_2_, *a*_3_, *a*_4_ and *a*_5_ were obtained by minimizing the root mean square error (RMSE = $\sqrt{{\left(g_{act}-g_{exp}\right)}^{2}}$) with respect to experimental data (*g*_*exp*_) using the Curve Fitting Tool from MATLAB (Version R2020b, MathWorks, Natick, MA, USA).

#### 2.2.2. Experimental characterization

To determine the coefficients of *p*_*sr*_, a closed-, non-actuated loop filled with a glycerine-water (40–60%) mix mimicking the viscosity of blood (Figure 3A) was subjected to the contraction of the soft robot at 60 bpm with *p*_*act*_ at 6, 7, 8, 9, 10, 11, and 12 psi. The duration of actuation *t*_*act*_ was set to 300 ms which was previously found to be most effective in generating pressure between different parts of the cardiac cycle (35). We used 2.5 cm diameter silicone tubing (high-temperature silicone rubber, durometer 35A) with 1 mm wall thickness and total length of 2 m. A one-directional flow valve was used to inhibit back flow in the loop. The pressure *p*_*aorta*_ was tuned to 50, 60, 70, 80, 90, 100, 125, and 150 mmHg by changing the glycerine-water mixture volume in the loop *via* a syringe (Figure 3). No significant kinking of the silicone vessel wall was observed during actuation of the soft robot at *p*_*aorta*_ = 75*mmHg* as measured my magnetic resonance imaging (Supplementary material). Pressure and flow were measured using a pressure transducer (Mikro-Tip Catheter Transducer SPR-350s, Millar Inc., Houston, Texas, USA) and flow probe (ME14PXL294, Transonic Systems Inc., Ithaca, New York, USA). The measured data was recorded using the LabChart software (ADInstruments, Dunedin, New Zealand) and the PowerLab 8/35 combined with the Power Bridge Amp hardware (ADInstruments, Dunedin, New Zealand).

### 2.3. Coupling the patient-specific and soft robot lumped parameter models

The formulation of *p*_*sr*_ was validated through RMSE analysis (Supplementary material). After validating the patient-specific lumped parameter model through comparison with hemodynamic data, the soft robot lumped parameter element was added between the first two ascending aorta vessel elements of the patient-specific cardiovascular model. A difference in capacitance and resistance between the experimental set-up and the patient leading to a different peak *p*_*sr*_. We solved this by multiplying *p*_*sr*_ by a correcting factor. The factor was determined for *p*_*aorta*_ = 100 mmHg. The resulting coupled system was solved numerically with a time step of 0.1 ms over 10 cardiac cycles.

## 3. Results

### 3.1. Patient-specific model

The patient-specific lumped parameter model was validated through a comparison with key hemodynamic parameters of the *in vivo* patient data acquired through magnetic resonance imaging and echocardiogarphy (Table 1). Good agreement between the patient-specific model and patient data was achieved (relative error) for all important hemodynamic parameters such as diastolic aortic pressure (+7%), systolic aortic root pressures (+1%), peak flow (+1%), cardiac output (+0%), flow split (1%), end-diastolic volume (+3%) (Table 1).

**Table 1:** Patient data compared with the patient-specific closed-loop computational model (abs, absolute; press., pressure).

	**Patient**	**Model**	**Δ (abs)**	**Δ (%)**
Heart rate (bpm)	60	60	0	0
Diastolic aortic root press. (mmHg)	68	73	+5	+7
Systolic aortic root press. (mmHg)	150	147	−3	−2
Peak flow (mL/s)	266	267	+1	+1
Cardiac output (L/min)	5.17	5.17	0	0
Flow split (upper/lower body) (%)	29/71	30/70	1	1
End diastolic LV volume (mL)	116	119	+3	+3
End systolic LV volume (mL)	36	34	−2	−8
End diastolic LV press. (est.) (mmHG)	16	16	0	0

**Figure 4:**
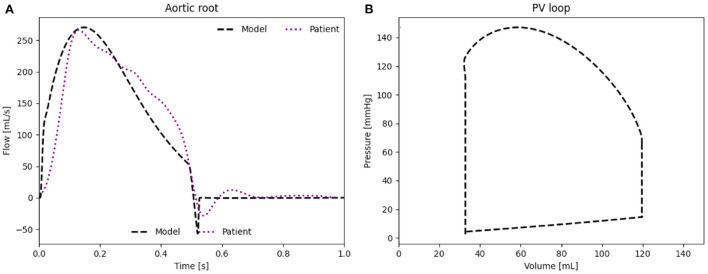
**(A)** Flow rate through the aortic root induced by the LV. **(B)** PV loop of the LV of the patient-specific model.

The waveform as well as the peak flow rate of the model, presented in Figure 4A, match well with the flow measured in the HFpEF patient. The RMSE between the *in vivo* flow data and the patient-specific model calculated over a full cardiac cycle is 36.1 mL/s. The RMSE can be partly explained through the small time shift between both flow peaks. The modeled PV loop of the LV (Figure 4B) is composed of an isovolumetric pressure increase and decrease phases during LV contraction and relaxation as well as a small pressure increase during diastole. The flow splits into the main branches of the cardiovascular system are in line with the *in vivo* flow splits presented in literature (36). Moreover, the PV loop is closed, proving that the computational model has stabilized and is running identical cycles as it has reached a time periodic state.

### 3.2. The impact of the soft robotic device prototype on the patient

We present the influence of the actuator delay δ*t*_*act*_ between 50 and 950 ms with a step of 50 ms and the different *p*_*sr*_ between 6 and 12 psi with a step of 2 psi on the patient where peak systolic pressure reduction, lower stroke work, and higher cardiac output are the key indicators for beneficial actuation of the soft robot.

**Figure 5:**
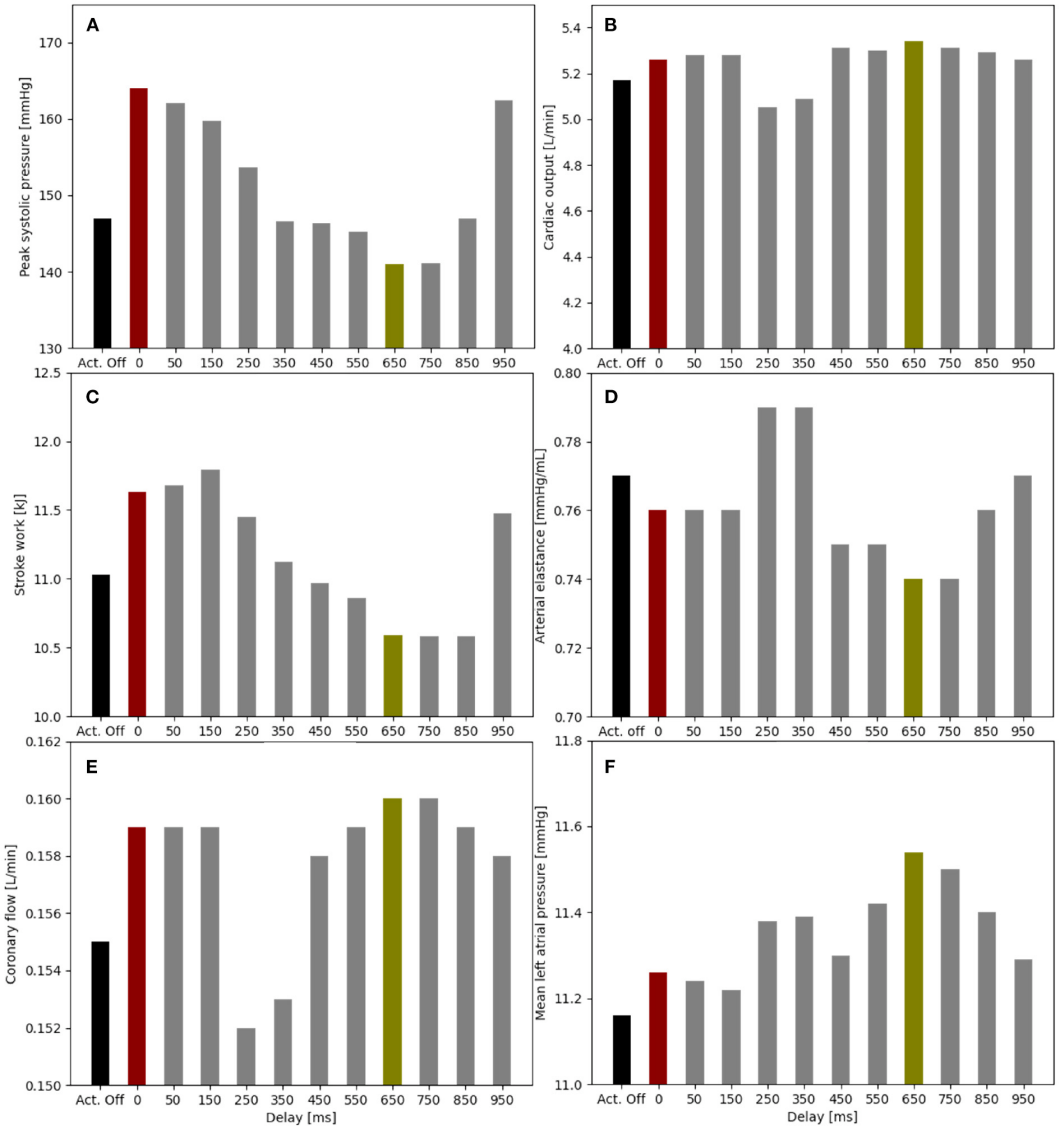
Impact of the soft robot delay on peak systolic pressure **(A)**, cardiac output **(B)**, stroke work **(C)**, arterial elastance **(D)**, coronary flow **(E)**, and mean left atrial pressure **(F)** (*p*_*act*_ = 12 psi). Optimal case for delay at 650 ms (green), baseline for actuator off (Act. Off) (black) and worst case for delay at 0 ms (red).

An actuation delay of 650 ms results in maximal peak systolic pressure reductions (141 vs. 147 mmHg), allows for the highest cardiac output (5.34 vs. 5.17 L/min) and the lowest stroke work (10.6 kJ compared to 11.8 kJ) compared to baseline (Figure 5). In this optimal case, the arterial elastance *EA* (*EA* = *ESP*/*CO*, where *ESP* is the end systolic pressure) is also reduced compared to baseline. The delay of 650 ms together with 300 ms actuation time mean that the soft robot relaxes shortly after the start of the ventricular contraction. However, the left atrial pressure increases upon actuation (11.5 mmHg compared to 11.2 mmHg). On the contrary, the worst case actuation was found to be at a delay of 0 ms and thus occurring simultaneous to the ventricular contraction creating higher peak systolic pressure (164 vs. 147 mmHg) and stroke work (11.8 vs. 11.1 kJ).

**Figure 6:**
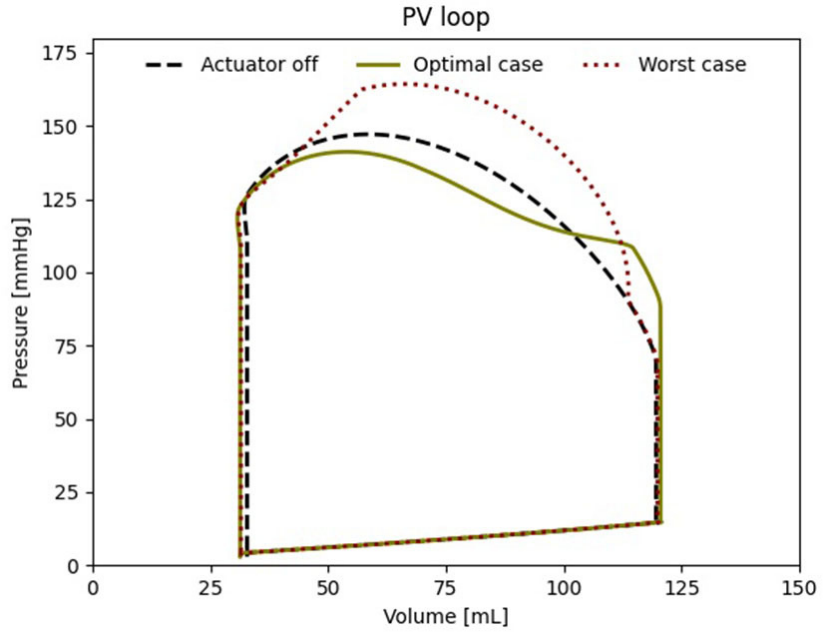
Left ventricular PV loop for the best case (650 ms delay), the worst case (000 ms delay) and baseline (*p*_*act*_ = 0 psi).

**Figure 7:**
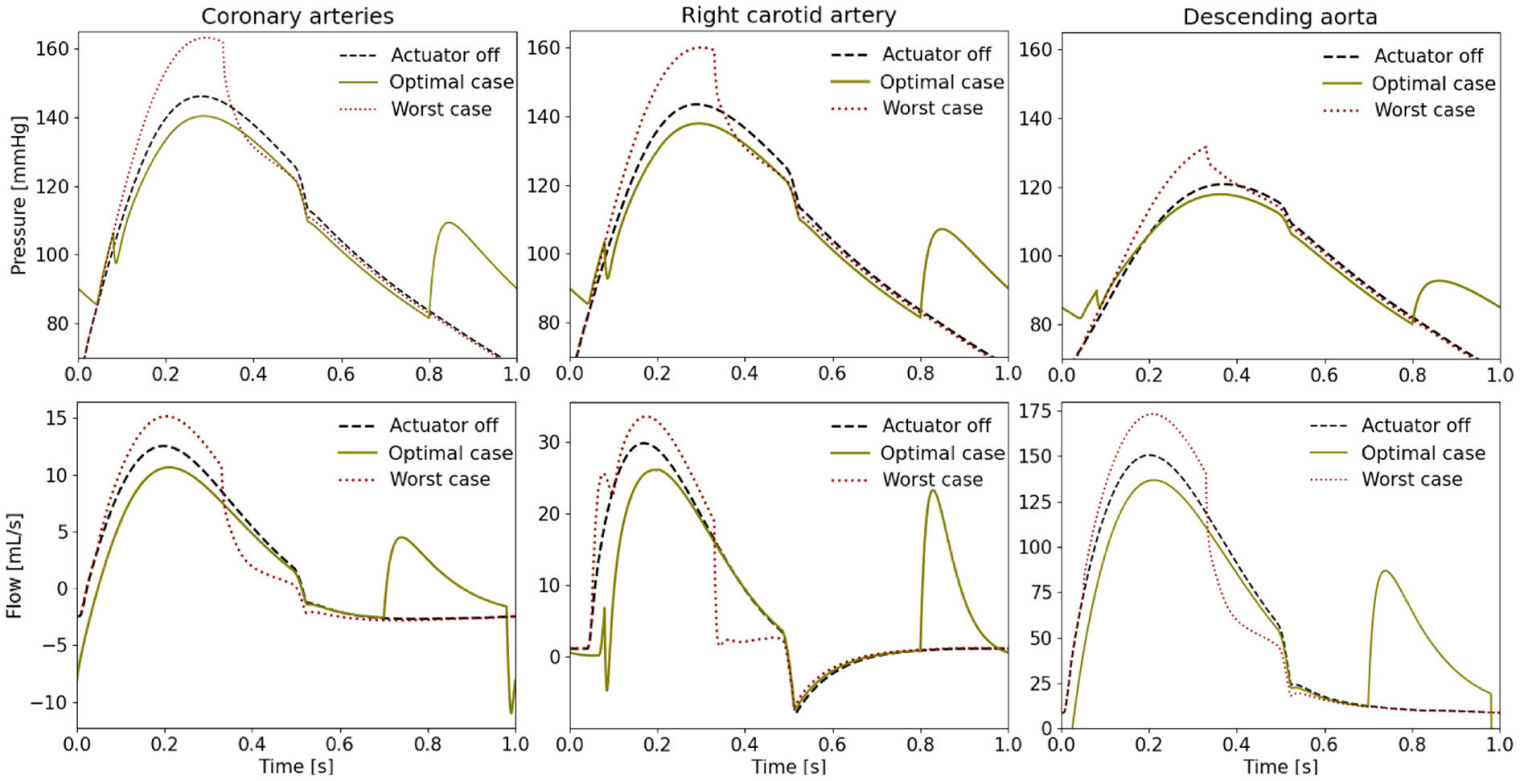
Pressure and flow waveforms in the coronary and carotid arteries and the descending aorta (*p*_*act*_ = 12 psi). All the measurements presented in this figure are made at the interaction of the last vessel element of the branch of interest and its proximal RCR element.

When comparing the PV loops of the optimal and baseline settings we see that the diastolic filling volume is increased while the end systolic volume is decreased resulting in a larger stroke volume. Moreover, the peak systolic pressure is decreased (Figure 6). A second pressure and flow peak during diastole can be seen in all branches of the aorta in the optimal case (Figure 7). The pressure peak decays slowly over 0.3 s while the flow peak reaches its maximum after 0.1 s before disappearing after 0.2 s. The highest device-induced diastolic pressure increase at *p*_*act*_ = 12 psi is seen in the coronary arteries (25 mmHg), followed by the carotid arteries (23 mmHg) and the descending aorta (11 mmHg). While, the overall cardiac output increased, this diastolic support did only lead to a small increase in coronary flow. In the worst case, the pressure and flow created by the soft robot adds up on top of the systolic activity of the heart, further increasing the afterload.

The flow splits toward the separate parts of the upper body are not known for the patient but their modeled values are presented in Table 2. The flow splits to the peripheral vessels remain similar to baseline in the optimal case. The soft robot increased absolute flows into each branch compared to baseline. The largest increase in flow is directed toward the descending aorta increasing the percentage of received flow. The absolute flow to the coronaries increased by six percent when the patient is supported by the extra-aortic support device.

**Table 2:** Flow splits into the different parts of the cardiovascular system in the patient-specific model without and with support (delay = 650 ms) at 60 bpm, (CO, cardiac output; Abs., absolute).

	**Actuator off**		**Optimal case**	
CO (L/min)	5.17		5.34	
**Vessel**	**Abs. flow (L/min)**	**%**	**Abs. flow (L/min)**	**%**
Descending aorta	3.63	70.27	3.74	70.37
Coronaries	0.16	3.00	0.16	3.00
Right subclavian	0.38	7.45	0.39	7.42
Right carotid	0.47	9.18	0.49	9.15
Left subclavian	0.25	5.25	0.28	5.23
Left carotid	0.25	4.86	0.26	4.84

### 3.3. The effect of actuator pressure

**Figure 8:**
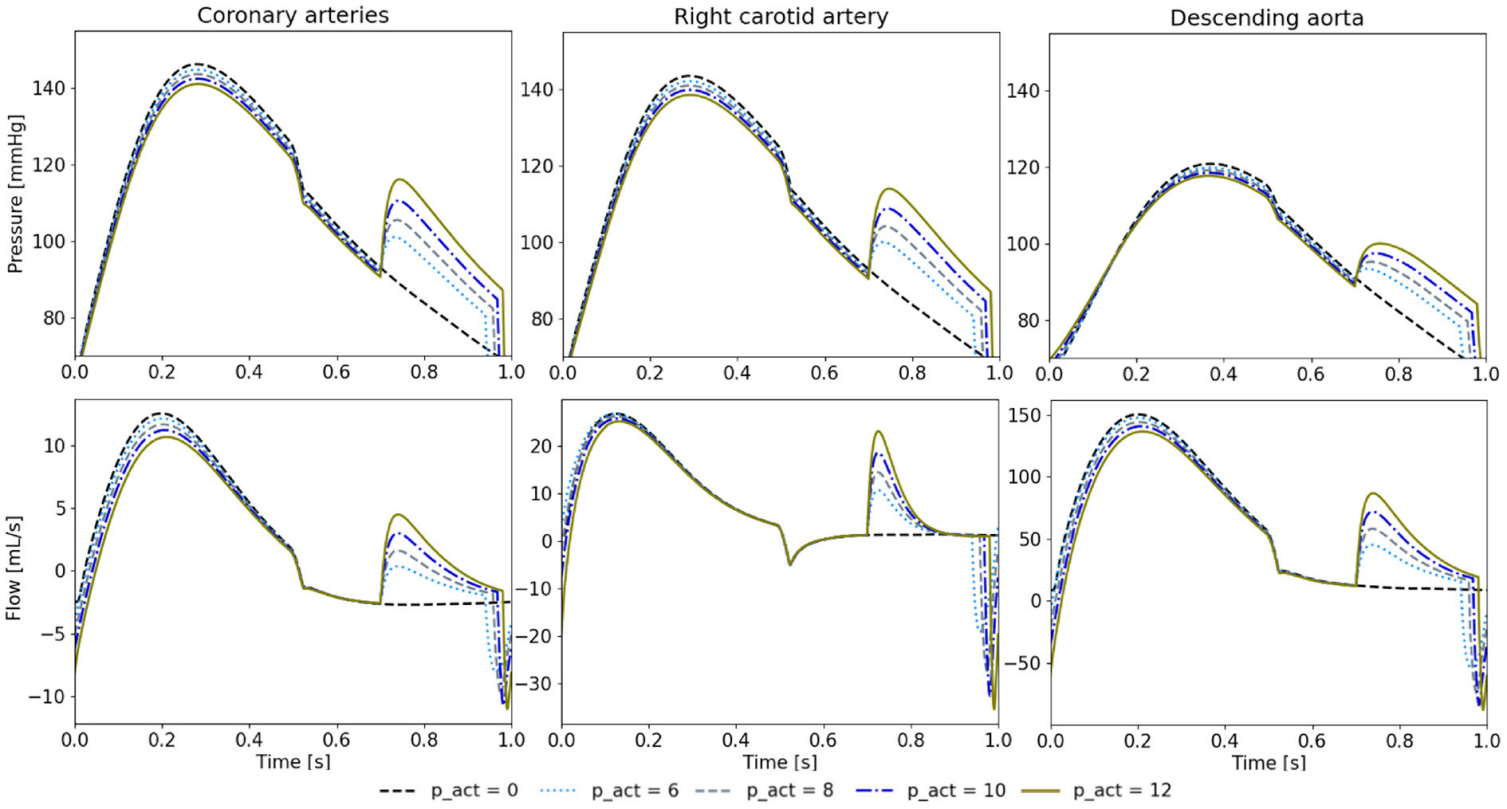
Pressure and flow waveforms in the coronary and carotid arteries and the descending aorta for 650 ms of delay and different *p*_*sr*_ in psi. All the measurements presented in this figure are made at the interaction of the last vessel element of the branch of interest and its proximal RCR element.

We note a higher diastolic pressure increase during diastole with a higher *p*_*sr*_ (Figure 8). In turn, this also leads to stronger reduction of peak systolic pressures. Similarly, the cardiac output increases with the higher *p*_*sr*_ (5.22 L/min at 6 psi, 5.26 L/min at 8 psi, 5.29 L/min at 10 psi, and 5.34 L/min at 12 psi).

## 4. Discussion

We created a patient-specific lumped parameter model of a HFpEF patient and a soft robotic extra-aortic counterpulsation device prototype to study the physiological implications of varying operating conditions. Full tuning to the patient-specific flow dynamics was achieved. We found that if operated in the optimal setting, we could achieve lower systolic pressure, a lower stroke work, and slightly improved perfusion of the coronaries at the cost of higher left atrial pressures.

This study evaluated a treatment strategy for HFpEF in a patient-specific model tuned to an individual subject. Previous work on modeling device-based treatment strategies in HFpEF have focused on the use of averaged population characteristics and grouping based on hemodynamic phenotypes (11–14, 37). Burkhoff et al. extensively modeled four distinct phenotypes of HFpEF. The paper acknowledges that the group of patients with HFpEF induced by hypertension is highly variable in its hemodynamic characterization (11). We extended the prior population based approaches using an open-source computational framework to re-create a patient-specific hemodynamic model using the open-source framework Simvascular.

The device modeling was inspired by previous work on modeling counterpulsation devices in lumped parameter and 1D models (38, 39). Our approach informed the device model directly from an experimental analysis of the studied counterpulsation device prototype. Combining the model of the device prototype and patient-specific model allowed for patient-specific simulations of their interaction, which is critical given the number of different pathophysiologies found in HFpEF. Our simulation allows one to tune the settings of any potential device to the needs of a specific patient. In the future, the computational model could also allow to predict treatment effects prior to implantation.

Using our simulation, we evaluated the effect of counterpulsation on the hemodynamics in HFpEF at different timings. When actuated during optimal setting actuation (*p*_*act*_ = 12 psi, delay = 650 ms), an additional diastolic pressure and flow waveform is observed in all cardiovascular branches, which is inline with previous studies (34). The effect on systolic aortic pressure is similar to previous *in vivo* studies about IABPs and the C-Pulse System, however the cardiac output is increased to a much lesser extent (18, 21, 39, 40). While the effect on decreasing aortic pressure is similar, the soft robotic device prototype covers a significantly smaller aortic arch length then the C-pulse system. The small width of the actuator could allow for serial integration of multiple actuators in the future. Finally, we observed increased diastolic filling volume allowing for a larger stroke volume, however this comes at the cost of a higher left atrial pressure. In our simulation, the device is able to reduce the load on the heart, evidenced by the reduced stroke work.

The worst case actuation points toward the risks of the counterpulsation device with increased peak systolic pressure. A systolic actuation of the device can lead to an increase of peak systolic pressure by 17 mmHg, while the stroke work of the native heart increases by 0.7 kJ compared to baseline. The soft robotic actuator relies on accurate real-time sensing of the ventricular contraction to avoid harming the cardiovascular system, as has been shown in other pulsating soft robotic devices. While, the existing approaches to trigger IABPs might be useful for triggering of a soft robot, our results point toward the risks of pulsating technologies. In contrast to IABPs, the extra-aortic approach mitigates risks associated with infection and thromboembolic considerations, while several other risk factors will need to be addressed such as long-term functioning of the device as well as implications for the aortic valve.

We were also able to show the importance of the chosen *p*_*act*_ in tuning the effect of the soft robotic device prototype on the patient's physiology. A higher *p*_*act*_ led to lower systolic peak pressures as well as higher cardiac output. The ability to tune the impact allows for scenario-specific adaptation such as rest and exercise but also for gradual increase in support to the heart. Furthermore, a bridge to recovery application can be envisioned through support modulation. Based on the similarity of the flow waveforms between the soft robot and IABPs, no impact on cerebral function is expected (41).

Clinical considerations: The usefulness of counterpulsation strategies is highly debated (35). While IABP are used to bridge patients with HFrEF effectively, the underlying physiological mechanism remain unclear. The current recommendation made by the American College of Cardiology, the American Heart Association, and the Heart Failure Society of America is to lower the systolic blood pressure (SBP) under 130 mmHg for HFpEF patients with hypertension (42). However, Zouein et al. have shown antihypertensive medication to be ineffective in diminishing mortality in patients with HFpEF (4). If lowering systolic pressure (afterload) is beneficial in HFpEF, our study suggest that extra-aortic counterpulsation could be beneficial. However, the design of the current device prototype requires further optimization in order to displace sufficient volume.

Limitations: The study constitutes a framework for patient-specific hemodynamic modeling of device based treatment. The cohort of patients will need to be increased to cover a larger variety of patients as well as the heterogeneity in pathophysiology found in HFpEF. Moreover, the simulation currently lacks integration of the autoregulatory control mechanisms such as the baroreflex as well as coronary blood flow regulations. Furthermore, the effect of the silicon vessel on the evaluation of the soft robots was not specifically addressed in this work. Ongoing experimental work and *in-vivo* trials with the soft robotic actuator will enable further validation of the findings of the lumped parameter model presented.

## Data availability statement

The original contributions presented in the study are included in the article/Supplementary material, further inquiries can be directed to the corresponding author/s.

## Ethics statement

The studies involving human participants were reviewed and approved by IRB Veteran Association Palo Alto. The patients/participants provided their written informed consent to participate in this study.

## Author contributions

MA, DE, SD, and IC contributed to conception and design of the study. IC organized the data acquisition and provided clinical context. MA and SD performed the experiments and wrote the first draft of the manuscript. JP and AM provided access to the original code. All authors contributed to manuscript revision, read, and approved the submitted version.

## Funding

SD received funding from the Swiss National Research Foundation P2EZP2_188964. SD, IC, and DE received funding from the NIH grant RO1 HL131823. AM and JP received funding from NIH grants R01EB029362 and R01EB018302.

## Conflict of interest

The authors declare that the research was conducted in the absence of any commercial or financial relationships that could be construed as a potential conflict of interest.

## Publisher's note

All claims expressed in this article are solely those of the authors and do not necessarily represent those of their affiliated organizations, or those of the publisher, the editors and the reviewers. Any product that may be evaluated in this article, or claim that may be made by its manufacturer, is not guaranteed or endorsed by the publisher.
