# Supplementary material for: Framework for patient-specific simulation of hemodynamics in heart failure with counterpulsation support
Source: Front Cardiovasc Med. 2022 Aug 1;9:895291. doi: 10.3389/fcvm.2022.895291 (PMC9376255; doi:10.3389/fcvm.2022.895291)
Supplement: Supplementary file 1 [file Data_Sheet_1.PDF]

frontiers

Supplementary Material

1 SUPPLEMENTARY DATA

1.1 Supplementary Tables and Figures

1.1.1 Figure S1

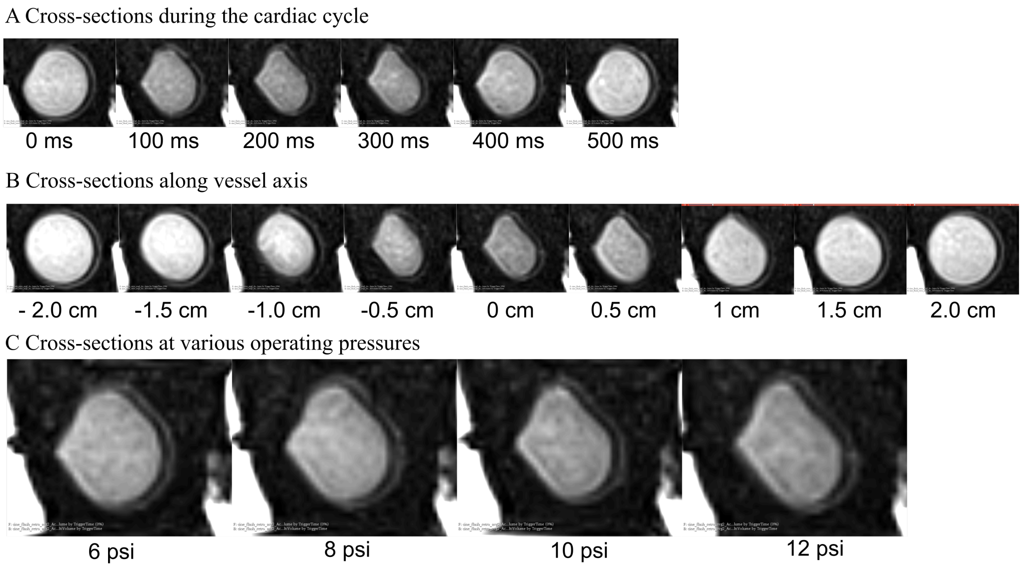

Figure S1. Deformation of silicone vessel wall upon inflation of that actuator, plotted (A) during the cardiac cycle, (B) along the vessel length, and (C) for various operating pressures. Images obtained using magnetic resonance imaging.

1.1.2 Figure S2

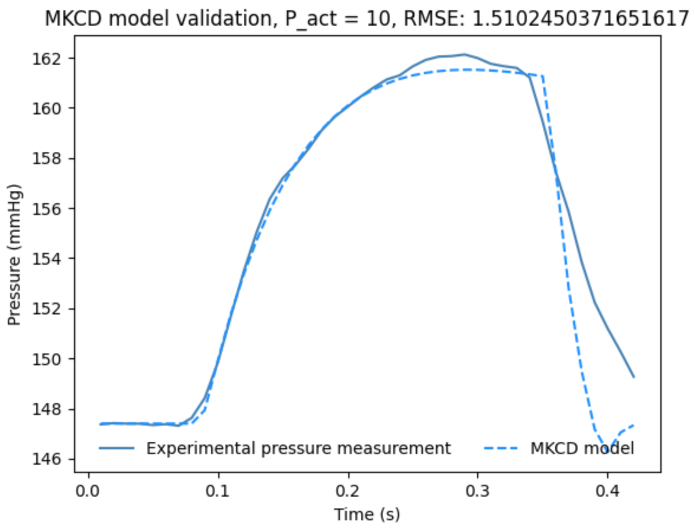

Figure S2. Comparison of P_{sr} with the measured pressure from the test bench (p_{act} = 10 psi.

RMSE (mmHg)		p_{aorta} (mmHg)								
		50	60	70	80	90	100	125	150	Avg.
p_{act} (psi)	6	0.67	0.74	0.64	0.55	0.58	0.74	0.87	0.54	0.67
	7	0.91	0.69	0.72	0.68	0.66	0.76	0.76	0.52	0.71
	8	1.37	0.89	0.92	0.91	0.91	0.88	1.06	0.68	0.95
	9	1.65	1.13	1.05	0.98	0.95	1.09	1.18	0.74	1.09
	10	1.62	1.25	1.19	1.16	1.08	1.06	1.11	0.75	1.15
	11	2.23	1.73	1.54	1.21	1.00	1.36	1.31	0.93	1.44
	12	2.20	1.90	1.80	1.47	1.51	1.54	1.49	1.09	1.62
	Avg.	1.52	1.19	1.12	0.99	0.98	1.06	1.11	0.75	1.09

Table S1. RMSE between P_{mkcd} and the experimental pressure waveforms. The average RMSE over all combinations of p_{aorta} and p_{act} is 1.090 (Avg.: Average RMSE).

1.1.3 Table S1

1.1.4 Table S2

Coefficient	Value
a1	2.253e-05
a2	-0.005474
a3	0.02852
a4	0.3111
a5	-0.0001239
RMSE	0.007599

Table S2. Coefficients for the $g_{act}(p_{act}, p_{aorta})$ function optimised to minimise the RMSE.

p_{act}	t_{on}	t_{off}
6	0.240	0.310
7	0.254	0.324
8	0.258	0.328
9	0.265	0.335
10	0.268	0.350
11	0.270	0.350
12	0.280	0.400

Table S3. t_{on} and t_{off} in the computational model of the MKCD.
